# Association between Environmental Exposures and Asthma among Children in King Williams Town, South Africa

**DOI:** 10.3390/diseases10040123

**Published:** 2022-12-05

**Authors:** Rasaq A. Yusuf, Phoka C. Rathebe, Thokazani P. Mbonane

**Affiliations:** Department of Environmental Health, Faculty of Health Sciences, Doornfontein Campus, University of Johannesburg, P.O. Box 524, Johannesburg 2006, South Africa

**Keywords:** asthma, environmental exposure, children, pollution, environmental tobacco smoke

## Abstract

The study aimed to assess the association between environmental exposure and asthma among children between 3 and 12 years old in King Williams Town, South Africa. A quantitative case-control study was conducted at Grey Hospital to assess the association between environmental exposure and asthma among children who reside in King Williams Town. Of the total 566 study participants, 50.5% (286) had asthma while 49.5% did not. Socio-demographic factors associated with asthma in children were being within the age group 9–12 years (OR 1.74, CI 95% 1.09–2.78) and India ethnicity (OR 0.20, CI 95% 0.08–0.48). Factors associated with asthma were weight within 25–35 kg (OR 1.64, CI 95% 1.11–2.42) and BMI within 15–20 (OR 4.80, CI 95% 2.80–8.22). Environmental risk factors associated with asthma were indoor exposure to tobacco smoke from mothers of the participants (OR 5.45, CI 95% 3.08–9.65) and from fathers (OR 4.37; CI 95% 2.77–6.90). Abstaining from eating seafood appeared to be protective from developing asthma (OR 0.01; CI 95% 0.00–0.05). The study found no significant association between outdoor environmental exposures and childhood asthma. The age of participant, weight, BMI, exposure to environmental tobacco smoke (ETS), and eating seafood had significant correlations with childhood asthma. Strengthening the evaluation of children healthcare and encouraging smoking cessation among parents could reduce exposure to environmental asthma triggers among children.

## 1. Introduction

Asthma is a respiratory disease that is highly prevalent in Africa and affects over millions of people globally. The symptoms of asthma were highly reported in low- and middle-income African countries between 1993 and 2013 due to various changes in environmental conditions, such as an increase in domestic lifestyles in urban areas and areas with greater industrial development [1]. In children, asthma is more severe due to the narrow and still-developing airways, as well as increased susceptibility to irritation from environmental agents [2]. A narrow and hyper-reactive airway coupled with complex nose breathing patterns among neonates and infants can influence the development of asthma. Neonates and infants have weak lung muscles coupled with higher basal metabolic oxygen requirements, immature control of breathing and different airway mechanics [3], which contribute significantly towards the development of childhood asthma. These factors play a significant role in cell growth, maturation and a body’s susceptibility to various environmental agents.

Furthermore, Saadeh and Klaunig [4] suggested an association between asthma development, nutrition, and respiratory system susceptibility, as well as residential history; this association can also be found in African countries due to poor nutrition and low socioeconomic status. Wjst and Boakye [5] found the prevalence of asthma in some African countries as—9.1% in Ethiopia, 15.8% in Kenya, 8.7% in Algeria, and 11.9% in Tunisia—to be much lower than in South Africa (20.3%). Childhood asthma is associated with the prevailing poverty and environmental circumstances among Africa countries [5]. Moreover, poor infrastructure to access healthcare systems, low socioeconomic status and high levels of airborne pollutants are among the highest contributors of childhood asthma and mortality in Africa [5,6]. A longitudinal study conducted among 39,907 singleton children (followed up between 1959–1976) by Chu et al. [7] suggested that women who were treated for pregnancy-related infections with penicillin or chloramphenicol had an increased risk of giving birth to asthmatic children.

In-utero exposure of unborn babies to certain medications also contributes to childhood asthma. The association between food allergies and childhood asthma was reported in the United States [8] where serum samples (404) were evaluated for a specific immunoglobin to common food allergies such as egg, milk, soya beans, peanut, wheat, and fish. Forty-four percent (44%) of study subjects showed evidence of sensitization (food-specific IgE ≥ 0.35 kU/L) to at least one food item, leading to increased asthma healthcare and medication use [8].

In South Africa, both in rural and urban settings, the prevalence of childhood asthma continued to increase due to rapid urbanization and uncontrolled environmental pollution. In the context of environmental pollution from noxious gas emissions, it was revealed that the Vaal Triangle area of South Africa was the most polluted area with nitrogen dioxide due to a heavy industrial presence in the area [9]. Likewise, the presence of large industrial and transportation networks in the South Durban Basin in the province of Kwa Zulu-Natal is a major concern, as it produces air pollution and releases noxious chemicals and large amounts of sulfur dioxide into the atmosphere [10]. Another area known for gaseous emissions in the country is around Secunda in the province of Mpumalanga [11]. This area is dotted with coal power plants that release sulfur dioxide and other harmful gases into the environment. Residents around these hot spots of air pollution in South Africa are constantly exposed to air pollutants, which results in a higher likelihood of developing various cardiorespiratory diseases. In the Eastern Cape province of South Africa, in the Buffalo City Metro Health District (BCMHD), a total of 171 deaths were recorded between 2010 and 2015 due to childhood asthma and asthma-related events [12]. The ongoing concerns of air pollution, illegal dumping of waste, outdoor burning of refuse, and indoor use of solid fuels, might be imputable factors for childhood asthma attacks and deaths within the locality.

The aim of this study was to assess the association between environmental exposures and asthma among children between the ages of 3 and 12 years old in King Williams Town, South Africa.

The following objectives were pursued:

(i) To examine the association between asthma among participants by age and gender;
(ii) To determine the relationship between childhood asthma among participants according to weight, height, and body mass index (BMI);
(iii) To assess symptomatology of asthma among children;
(iv) To determine the association between environmental exposures and asthma among children.

## 2. Materials and Methods

### 2.1. Study Design

A quantitative, cross-sectional design was adopted to assess the association between environmental exposures and asthma among children who are 3–12 years old in King Williams Town, South Africa. The study assessed exposure to environmental agents among the case group of asthmatic children and among the control group of children without asthma of similar age to the cases. The study was conducted in a district hospital in King Williams Town, South Africa.

### 2.2. Study Site/Area

The study took place in King Williams Town, which lies on the southeastern part of South Africa at an altitude of 405 m above sea level, −32.8808 latitude and 27.3945 longitude in the Province of Eastern Cape. The climate is classified as warm and temperate with average annual temperature of 18 °C and average annual rainfall of 600 mm [13]. The town is often dusty during the summer (December to February) season due to dust emanating from the surrounding untarred roads; moreover, the town is often dusty during autumn (June to August) due to pollen dust.

### 2.3. Study and Target Population

The total population of King Williams Town with the surrounding locations is 105,000 out of the total 55 million population of South Africa. The estimated total population of children (ages 12 and younger) in King Williams Town is 9500 [12]. The target population for the study comprised all children who were treated for asthma in King Williams Town—a peri-urban settlement. Participants were selected among children who attended Grey Hospital for asthma treatment during January–November 2021.

Prior to commencement of this study, ethical clearance was obtained in October 2020 (with subsequent renewal in January 2022) from the Faculty of Health Sciences Research Ethics Committee at the University of Johannesburg.

### 2.4. Sampling Strategy

A purposeful sampling method was used based on the select medical records that were available for children treated for asthma. Subsequently, a simple random sample was used to select medical records of children who were not treated for asthma. The total sample size for the study was 566: 286 asthma cases and 280 controls without asthma. The sample size of the study was determined using Epi Info 7.20, with 95% confidence intervals and 80% statistical power. For the unexposed group, a risk ratio of 2:2 and 25% as the percentage of outcome were used; in addition, for the exposed group, a 1.7 risk ratio, a 2.43 odds ratio, and 51% as the percentage of outcome were used. 

The initial intention of this study was to collect primary data from caregivers/parents/guardians of the eligible children who were going to be interviewed and respond to questions on behalf of their children. However, secondary data were used as an alternative due to the ongoing COVID-19 pandemic. Therefore, a questionnaire was designed by the researchers according to the ISAAC manual [14] to abstract data from the medical records of eligible participants.

### 2.5. Data Type

The variables measured in this study were: asthma as the dependent variable; environmental agents/exposures as independent variables (indoor, outdoor, allergens, in-utero, anaphylaxis, and physical exercise); and age (3–12 years old) as covariates. The details of variables are provided in Table 1.

### 2.6. Data Analysis

Data was analyzed using SPSS version 26.0 and variables were analyzed per study objective. Epi Info 7.20 was mainly used to calculate the crude odds ratios. Data were analyzed based on the study objectives:

Objective 1: The frequency distribution, cross tabulation, crude odds ratio, and confidence intervals were used to quantify asthma among participants by age and gender groups.

Objective 2: The mean, standard deviation, *t*-test, and *p*-value (0.05 significance level) were used to determine the relationship between childhood asthma among participants according to weight, height, and body mass index (BMI).

Objective 3: The frequency distribution, cross tabulation, crude odds ratio, and confidence intervals were used to assess symptomatology of asthma among study participants.

Objective 4: The frequency distribution, cross tabulation, crude odds ratio, and confidence intervals were used to quantify environmental exposure and asthma among participants according to indoor, outdoor, and other environmental agents.

## 3. Results

The socio-demographic characteristics of the participants were gender, age, and ethnicity. The numbers and raw percentages are presented for each socio-demographic characteristic.

### 3.1. Distribution of Study Participants by Socio-Demographics and Other Variables

**Figure 1:**
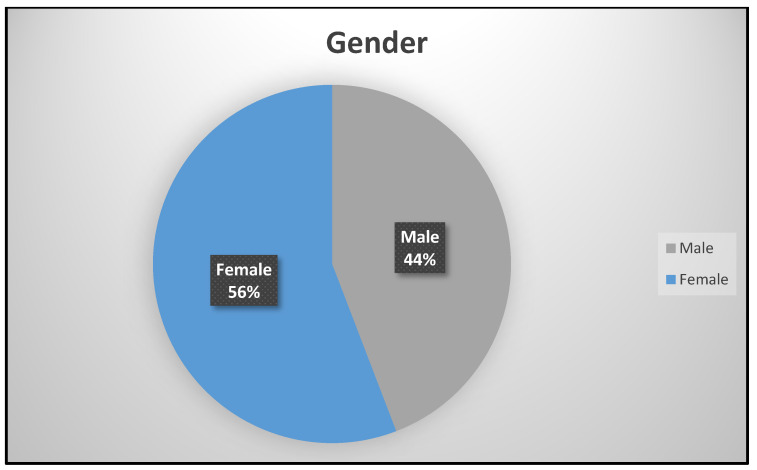
Gender distribution.

Gender was dichotomized as male (250; 44%) and female (316; 64%) in Figure 1. There were more females who attended the study site for asthma management and other services than males. This is attributed to the fact that there are more females in King Williams Town than males as is reported in the population census [9]. The age of the participants in years were grouped into Group 1 (155; 27.4%), Group 2 (277; 48.9%), and Group 3 (134; 23.7%), respectively.

**Figure 2:**
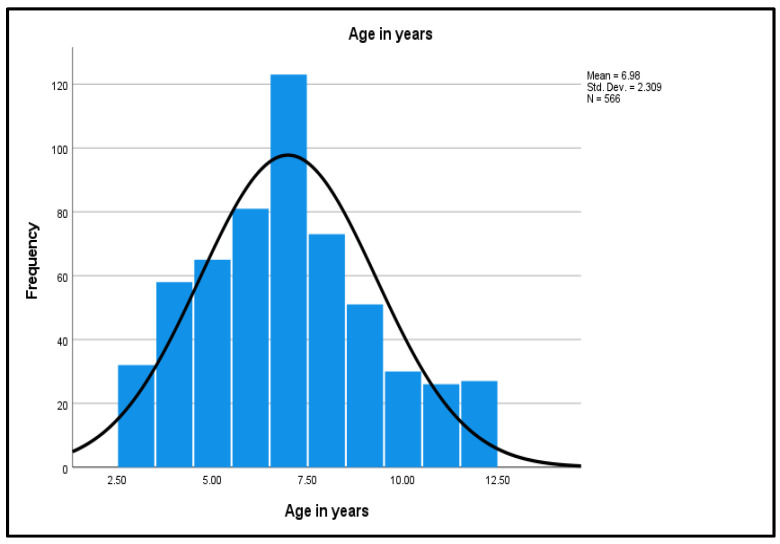
Normality Curve on the Age Distribution.

As shown in Figure 2, the age distribution shows a normal distribution curve, a unimodal peak, and a symmetric distribution with a center that lies between 5.0 to 10.0, with no outliers.

The mean (*M*) age of 6.98 was interpreted as 7 years. The small standard deviation (*SD*) of 2.31 reflects that the age values of the study dataset are close to the mean age. Weight of the participants ranged from a minimum of 14 kg to the maximum of 51 kg. The continuous variable was categorized into three groups: the lowest weight to 25 kg (389, 68.7%); 25–35 kg (140, 24.7%); and 36 kg to the highest weight (37, 6.5%). The highest frequency group was the lowest weight group (25 kg, 68.7%). Participants’ heights ranged from 0.93 m to 1.52 m. Height was recoded into three groups: the lowest height to 1.15 m (173, 30.6%); 1.16–1.40 m (347, 61.3%); and 1.41 m to the highest height (46, 8.1%). The highest frequency group was the middle height group (1.16–1.40 m) with 347 participants (61.3%). The lowest BMI was 11.89 and the highest BMI was 23.37 kg/m^2^. Subsequently, BMI was grouped into three groups: the lowest BMI to 15 (90, 15.9%); BMI 15–20 (473, 83.6%); and BMI above 20 (3, 0.5%). The highest frequency group was the middle BMI group (15–20) at 473 (83.6%).

### 3.2. Environmental Agents and Asthma among Participants by Age and Gender

The socio-demographic characteristics were presented with regards to having asthma or not having asthma. Crude analyses were performed using EPINFO 7.2 to enable visualization of the data. The numbers, raw precents, crude odds ratio, and 95% confidence intervals (CI) were presented for each socio-demographic characteristic by asthma status. Table 2 presents the distribution and crude odds ratios of participants by socio-demographic characteristics.

Among the age subgroup 9–12 with 134 (23.7%) total participants, 79 (59%) had asthma while 55 (41%) did not have asthma. The odds ratio (OR) of 1.74, with the confidence intervals (CI) of 1.09–2.78, was statistically significant. Greater probability of asthma among this subgroup was observed; thus, there an association exists.

In the subcategory of weight 25–35 kg with 140 (24.7%) participants, 84 (60%) had asthma while 56 (40%) did not have asthma. Statistical significance was found with an OR of 1.64 and a CI of 1.11–2.42 (Table 2), suggesting a greater probability of asthma among this subcategory.

The total number of participants with BMI 15–20 was 473 (83.6%). Out of these, 266 (56.2%) had asthma while 207 (43.8%) had not been diagnosed with asthma. In this subcategory, an OR of 4.80 was statistically significant with a CI of 2.80–8.22, indicating an association as well as a higher chance of incidence of asthma among these subcategories.

### 3.3. Environmental Agents and Childhood Asthma According to Weight, Height, and Body Mass Index (BMI) Participants

Descriptive statistics of continuous data for weight, height, and BMI were examined with the *t*-test to compare the means of the two groups. Participants with asthma (*M* = 7.26, *SD* = 2.38) and participants without asthma (*M* = 6.7, *SD* = 2.21) differed significantly on age (*p* < 0.004). When considering weight, asthma among the participants (*M* = 24.62, *SD* = 6.24) did not differ from participants without asthma (*M* = 23.44, *SD* = 10.04) (*p* ˃ 0.092). There was a significant difference among participants with asthma and those without asthma on height (*p* ˂ 0.022). No significant differences were observed among participant with asthma and participants without asthma (*M* = 15.90, *SD* = 3.23) for BMI (*p* ˃ 0.212).

### 3.4. Symptomatology of Asthma among Study Participants

Selected asthma symptoms were presented with regards to having asthma or not having asthma in Table 3. The numbers, raw precents, crude odds ratio, and 95% CI were presented for each asthma symptom by asthma status. The majority of participants (551, 97.35%) showed no wheezing that limit speech. Though statistical significance exists between those who reported asthma and non-asthma participants, a decreased probability of asthma with a protective association was observed (OR 0.15, CI 0.03–0.68). Among the study participants, 278 reported not coughing at night (49.1%) with 15 (5.4%) of them diagnosed with asthma while the remaining participants 263 (94.6%) had no asthma.

### 3.5. Indoor Environmental Exposure and Contact with Animal among Study Participants

There were reports of indoor exposure to different environmental agents among the participants as shown in Table 4. Smoke from cooking fuels (coal, wood, kerosene, and gas); exposure to cigarette smoke from the mother, the father, and others in the same household who smoke; and exposure to pets such as cats and dogs, as well as exposure to farm animals such as cows, goats, and donkeys, were reported. Eighty-seven (15.37%) of mothers or female guardians of participants smoked cigarettes. A total of 71 (81.6%) children of these women developed asthma while 16 (18.4%) had did not develop asthma. The OR of 5.45 with a CI of 3.08–9.65 was statistically significant, indicating an association with greater probability of asthma among this subgroup. A total of 125 (22.08%) children had fathers who smoked cigarettes, of which 96 (76.8%) had asthma while 29 (23.2%) had no asthma. The OR of 4.37 with a CI of 2.77–6.90 among the subgroup was statistically significant. There was a greater probability of asthma among participants who were exposed to smoke from their fathers, and, thus, there was an association between exposure to smoke from fathers and incidence of asthma among participants.

### 3.6. Outdoor Environmental Exposure among Study Participants

Various outdoor environmental agent exposures were reported among the participants: dust from children’s playgrounds, a dusty road, transport-related air pollution (TRAP), and pollen grain as shown in Table 5. There was no level of statistical significance reported among exposed groups in relation to outdoor environmental exposure (TRAP, grass [*axonopus*], and pollen) and asthma among participants.

### 3.7. Exposure to Other Environmental Agents among Study Participants

Other environmental agent exposures reported by study participants included foods such as meat, seafood, fruits, vegetables, pulses, cereals, pasta, rice, butter, margarine, nuts, potatoes, eggs, fast food, food additives, and other food types; medicine (paracetamol and antibiotics such as ampicillin, petercillin, co-trimoxazole, metronidazole, and other antibiotics); and in-utero exposure to paracetamol and antibiotics. All of these agents were not statistically significant, except for seafood, consumption of food with food additives, as well as children receiving paracetamol. Table 6 presents distribution and crude odds ratios of participants by asthma and exposure to some other environmental agents that were mostly reported by participants who were treated for asthma at the study site.

Among 459 (81.1%) of the total 566 participants, 181 (39.4%) who never ate seafood or were not known to have eaten seafood developed asthma while 278 (60.6%) did not develop asthma. The OR of 0.01 with a CI of 0.00–0.05 was statistically significant, which suggested a decreased probability of asthma among participants who never or were not known to have eaten seafood, and the association was protective. Although not statistically significant, a decreased probability of asthma was observed among those who never ate food with food additives and children who received paracetamol.

## 4. Discussion

Participants aged 9–12 years were more likely to have asthma, using ages 3–5 as the reference group. This finding is consistent with Nair et al. [15]. In ages 9–12, a child’s airways are matured enough to elicit spirometry results, other lung function tests, and other asthma diagnostic procedures, which are necessary in order to make an appropriate asthma diagnosis. Older children are known to be more associated with asthma in general than younger children [16]. Participants aged 6–8 years were less likely to be diagnosed with asthma. This finding agrees with other studies [17,18], which showed that 20% of cases of asthma are diagnosed among children younger than 8 years. The difficulty in asthma diagnosis among this age group is a result of varied and nonspecific symptoms of asthma, making the diagnosis challenging. Essentially, an asthma diagnosis increases among children older than 8 years of age due to better diagnostic approaches.

In this study, the higher incidence of asthma among female participants may be related to the higher number of female participants enrolled. However, these findings contradict older studies with outcomes of higher prevalence in childhood asthma among boys than girls [19,20,21]. In this study, asthmatic children had increased body weight; this observation may be due to therapy (corticosteroids) for asthma. The finding is plausible because extra weight around the chest and abdomen might compress the lungs and make it more difficult to breathe [22,23]. Similar to the finding in this study, several epidemiological studies are consistent in the link between asthma and increased body weight [23,24,25]. Study participants with BMI 15–20 have a higher chance of developing asthma than the other BMI subcategories. This finding mimics the prevalence of asthma with increasing body weight as discussed in the preceding paragraph. Presently, there is growing evidence that increased BMI and obesity are associated with increased asthma and its morbidities [26,27], which accords well with the finding of this study.

Based on the finding of this study, participants who reported no wheezing are less likely to be diagnosed with asthma than participants with a history of wheezing. When airways become tightened, secondary to inflammation and congestion, breathing sounds like a characteristic whistling or squeaky. However, not all wheezing implies an asthma diagnosis. Nevertheless, wheezing is one of the most common signs of asthma. This is consistence with other studies [28,29] that identified wheezing as the most common symptom associated with childhood asthma. Children with breathing difficulties from upper and/or lower airways showing no wheezing may not necessarily have asthma. They should be further evaluated to rule out medical conditions that often present with dyspnea without wheezing, such as a foreign body in the airway, the common cold, sinusitis, pharyngitis, pneumonia, etc.

The incidence of asthma among participants who reported no night cough was lower than their counterparts who reported coughing at night. This finding is similar to other findings in the literature [29,30,31]. Children presenting to a family doctor with chest pathology without a wheeze and recurrent night cough are less likely to have asthma [32]. Nevertheless, an absence of a night cough among children cannot be used exclusively to rule out asthma without considering the presence or absence of other symptoms.

Participants from households where mothers or female guardians smoke cigarettes have a greater probability of developing asthma. These findings are consistent with previous studies. In a longitudinal case-control study [33], maternal smoking was associated with increased incidence of asthma among preschool children (OR 1.31, 95% CI 1.22 to 1.41). Furthermore, it was indicated that a parental smoking habit is also associated with wheezing and asthma among children younger than 12 years. This was substantiated by Zhuge et al. [34]. The researchers showed that environmental tobacco smoke (ETS) exposure is a risk factor for asthma and other respiratory health problems among children.

There are various outdoor environmental agent exposures reported among study participants: dust from children’s playgrounds, a dusty road, transport-related air pollution (TRAP), and pollen grain. However, there was no level of statistical significance reported in the study. The finding in this study is in contrast with [35], a systematic review where it was found that outdoor environmental exposures increase the risk of pediatric asthma. Certain outdoor environmental exposures (TRAP, carbon monoxide, pollen grain, and particulate matter (PM_2.5_)) were linked with asthma among toddlers and pre-teenage children. In addition, [36] estimated that over 30% of all childhood asthma cases could be invariably attributed to outdoor air pollution exposures (PM_2.5_) in 18 European countries among pre-teenage children.

Among study participants who never ate fish or seafood such as prawn, lobster, shellfish, oyster, calamari, etc. or were not known to have eaten seafood, there was a decreased chance of developing asthma than those who were exposed to seafood. Seafood, particularly shellfish, is known to cause allergies among children who have been introduced to seafood in the early stages of their adolescence [37]. This is because of inhalation of seafood muscle proteins and digestive enzymes such as trypsin that cause an allergic reaction or inflammation in the respiratory tract [38]. This type of allergy increases the likelihood of an asthma attack in children. However, in our study we did not consider the type of seafood consumed, which is a limitation, but we are inclined to believe that those who presented the risk of asthma due to consumption of seafood were introduced to it in their early adolescence stages and might have consumed shellfish. The risk of asthma among those who consumed seafood is incongruent with a systematic review [39], where the early introduction of fish into a children’s diet, with regular consumption of fish, was associated with improved asthma symptoms and a reduced risk among pre-teenage children compared to no fish consumption. Similarly, a La Trobe University clinical trial showed that eating fish, such as salmon, trout, and sardines, as part of a healthy diet can reduce asthma symptoms in children [40]. Nevertheless, there was a report of a severe asthma attack and anaphylaxis episode caused by sea urchin roe [41]. Putting our study’s findings on seafood and literature findings into perspective, it is inconclusive whether a seafood diet could be used as a reliable preventive strategy for childhood asthma. However, the introduction of seafood in the early developmental stages of children could reduce the risks of asthma.

## 5. Conclusions

The findings from this study showed that there is an association between pediatric asthma and environmental exposures (ETS and seafood). Moreover, there is an association between pediatric asthma and socio-demographic characteristics, body weight, BMI, and indoor ETS due to maternal and parental smoking. However, exposure of participants to outdoor environmental agents was not statistically significant in this study. Nevertheless, findings from this study denote advice on how childhood asthma could be prevented in King Williams Town, South Africa, by modifying exposure to certain amenable features. This could be achieved through concerted efforts between participants’ parents/caregivers and healthcare workers. Therefore, apart from a sole focus on a biomedical approach to childhood asthma management within, the need will be accentuated for opportunistic health promotion and childhood asthma prevention. The cross-sectional nature of this study is a limitation when considering the association between seafood and childhood asthma in the study area. Epidemiological studies are needed to validate the type of seafood, consumption frequency, and adolescent age; in addition, studies are needed to consider how underlying respiratory health conditions are potentially associated with the prevalence of asthma diagnosis and symptom severity.

## Figures and Tables

**Table 1:** Environmental agents and demographic variables assessed in this study.

Environmental Agents					
Indoor	Outdoor	Allergen	In-Utero	Anaphylaxis	Demographics
ETS	Pollen dust	Eggs	Penicillin	Bee sting	Sex/Gender Male and Female
VOCs	TRAP	Milk	Chloramphenicol	Medications: proton pump inhibitors	Age Group 1 (Ages 3–5) Group 2 (Ages 6–8) Group 3 (Ages 9–12)
Cooking smoke	Respiratory viral infection	Peanut	Paracetamol	-	Weight Lowest to 25 kg 25–35 kg 36–Highest
Dander	Ozone	Fish	Sulfadiazine-trimethoprim	-	Height Lowest–1.15 m 1.16–1.40 m 1.41–Highest
Dust mite	Industrial emissions, NO_2_, SO_2_	Crustaceans	Maternal alcohol abuse	-	Body Mass Index Lowest BMI–15 BMI 15–20 BMI above 20
Cockroach and rodent	Particulate matter; PM_2.5_, PM_10_	Wheat	Maternal use of tobacco (nicotine)	-	

ETS (environmental tobacco smoke); VOCs (volatile organic compounds); TRAP (transport-related air pollution); NO_2_ (nitrogen dioxide); SO_2_ (sulfur dioxide); PM (particulate matter); BMI (body mass index).

**Table 2:** Distribution and Crude Odds Ratios of Participants by Socio-demographic Characteristics.

	Total		Asthma		Without Asthma		Crude Odds Ratio	95% CI
Characteristics	N	%	N	%	N	%		
Total	566	100%	286	50.5%	280	49.5%		
Gender								
Male	250	44.2%	129	51.6%	121	48.4%	1.08	0.78–1.50
Female	316	55.8%	157	49.7%	159	50.3%	Reference	Reference
Age in years								
3–5	155	27.4%	70	45.2%	85	54.8%	Reference	Reference
6–8	277	48.9%	137	49.5%	140	50.5%	1.19	0.80–1.76
9–12	134	23.7%	79	59.0%	55	41.0%	1.74	1.09–2.78
Weight								
Lowest–25 kg	389	68.8%	186	47.8%	203	52.2%	Reference	Reference
25–35 kg	140	24.7%	84	60.0%	56	40.0%	1.64	1.11–2.42
36–Highest	37	6.5%	16	43.2%	21	56.8%	0.83	0.42–1.64
Height								
Lowest–1.15 m	173	30.6%	73	42.2%	100	57.8%	0.67	0.35–1.29
1.16–1.40 m	347	61.3%	189	54.5%	158	45.5%	1.10	0.59–2.03
1.41–Highest	46	8.1%	24	52.2%	22	47.8%	Reference	Reference
Body Mass Index								
Lowest BMI–15	90	15.9%	19	21.1%	71	78.9%	Reference	Reference
BMI 15–20	473	83.6%	266	56.2%	207	43.8%	4.80	2.80–8.22
BMI above 20	3	0.5%	1	33.3%	2	66.7%	1.87	0.16–21.72

BMI (body mass index).

**Table 3:** Distribution and Crude Odds Ratios of Participant according to Asthma Symptoms.

	Total		Asthma		Without Asthma		Crude Odds Ratio	95% CI
Characteristic	N	%	N	%	N	%		
Wheezing that limits speech								
Yes	15	2.6%	13	86.7%	2	13.3%	Reference	Reference
No	551	97.4%	273	49.5%	278	50.5%	0.15	0.03–0.68
Cough at night								
Yes	288	50.9%	271	94.1%	17	5.9%	Reference	Reference
No	278	49.1%	15	5.4%	263	94.6%	0.01	0.01–0.10

**Table 4:** Distribution and crude odds ratios of participants by asthma, indoor environmental tobacco smoke, and contact with animal.

	Total		Asthma		Without Asthma		Crude Odds Ratio	95% CI
Characteristic	N	%	N	%	N	%		
Child’s mother or female guardian smokes cigarettes								
Yes	87	15.4%	71	81.6%	16	18.4%	5.45	3.08–9.65
No	479	84.7%	215	44.9%	264	55.1%	Reference	Reference
Child’s father or male guardian smokes cigarettes								
Yes	125	22.1%	96	76.8%	29	23.2%	4.37	2.77–6.90
No	441	77.9%	190	43.1%	251	56.9%	Reference	Reference
Household source of fuel for cooking								
Electricity	142	25.1%	74	52.1%	68	47.9%	0.95	0.33–2.77
Gas	15	2.7%	8	53.3%	7	46.7%	Reference	Reference
Open flame	409	72.3%	204	49.9%	205	50.1%	0.87	0.31–2.45
Pet at child’s home during child’s first year of life								
Yes	59	10.4%	28	47.5%	31	52.5%	Reference	Reference
No	507	89.6%	258	50.9%	249	49.1%	0.78	0.43–1.40

**Table 5:** Distribution and Crude Odds Ratios of Participants by Asthma and outdoor environmental agents.

	Total		Asthma		Without Asthma		Crude Odds Ratio	95% CI
Characteristic	N	%	N	%	N	%		
Proximity of child’s residence to road								
Less than 10 m	8	1.4%	4	50%	4	50%	Reference	Reference
11–20 m	46	8.1%	22	47.8%	24	52.2%	0.92	0.20–4.12
21–50 m	76	13.4%	39	51.3%	37	48.7%	1.05	0.25–4.53
More than 50 m	436	77.0%	221	50.7%	215	49.3%	1.03	0.25–4.16
Exposure to grass, plant flower, pollen								
Yes	49	8.7%	49	100%	0	0%	Reference	Reference
No	517	91.3%	237	45.8%	280	54.2%	0.00	Undefined

**Table 6:** Odds of reporting asthma with estimated exposure to select other environmental agents assessed in this study.

	Total		Asthma		Without Asthma		Crude Odds Ratio	95% CI
Characteristic	N	%	N	%	N	%		
Discomfort while eating seafood								
Never or not known	459	81.1%	181	39.4%	278	60.6%	0.01	0.00–0.05
Once or twice per week	107	18.9%	105	98.1%	2	1.9%	Reference	Reference
Discomfort while eating food with food additives								
Never or not known	531	93.8%	264	49.7%	267	50.3%	0.58	0.29–1.18
Once or twice per week	35	6.2%	22	62.9%	13	37.1%	Reference	Reference
In the past 12 months, child received paracetamol								
Never or not known	428	75.6%	212	49.5%	216	50.5%	0.85	0.58–1.25
At least once in the past 12 months	138	24.4%	74	53.6%	64	46.4%	Reference	Reference

## Data Availability

Not applicable.
